# N-Terminal Truncated Myb with New Transcriptional Activity Produced Through Use of an Alternative MYB Promoter in Salivary Gland Adenoid Cystic Carcinoma

**DOI:** 10.3390/cancers12010045

**Published:** 2019-12-21

**Authors:** Candace A. Frerich, Hailey N. Sedam, Huining Kang, Yoshitsugu Mitani, Adel K. El-Naggar, Scott A. Ness

**Affiliations:** 1 Department of Internal Medicine, Division of Molecular Medicine, University of New Mexico Health Sciences Center, Albuquerque, NM 87131, USA; 2 Myriad Women’s Health, South San Francisco, CA 94080, USA; 3 Department of Internal Medicine, Division of Epidemiology, University of New Mexico Health Sciences Center, Albuquerque, NM 87131, USA; 4 Head and Neck Pathology, University of Texas MD Anderson Cancer Center, 1515 Holcombe Blvd, Houston, TX 77030, USAanaggar@mdanderson.org (A.K.E.-N.); 5 UNM Comprehensive Cancer Center, Albuquerque, NM 87131, USA

**Keywords:** transcriptional regulation, promoter switch, 5′RLM-RACE, alternative transcription start site, t(6;9)

## Abstract

Adenoid cystic carcinoma (ACC) is an aggressive salivary gland tumor that frequently displays perineural invasion and is often associated with translocations or overexpression of the *MYB* oncogene. Detailed analyses of *MYB* transcripts from ACC patient samples revealed that ACC tumors utilize an alternative *MYB* promoter, which is rarely used in normal cells or other tumor types. The alternative promoter transcripts produce N-terminally truncated Myb proteins lacking a highly conserved and phosphorylated domain, which includes the pS11 epitope that is frequently used to detect Myb proteins. In RNA-seq assays, Myb isoforms lacking the N-terminal domain displayed unique transcriptional activities, regulating many genes differently than full-length Myb. Thus, a regulatory pathway unique to ACC activates the alternative *MYB* promoter, leading to the production of a truncated Myb protein with altered transcriptional activities. This could provide new therapeutic opportunities for ACC patients.

## 1. Introduction

Adenoid cystic carcinoma (ACC) is an unpredictable and aggressive malignancy that most frequently occurs in the salivary gland and is treated by surgical rescission, but often results in local recurrences and late occurring, distant metastases [1,2,3,4]. ACC tumors have one of the highest rates of perineural invasion [5], a condition where tumor cells invade the surrounding nerves, which is associated with local tumor recurrence [6]. Recent studies have revealed previously unappreciated diversity amongst ACC tumors, with gene expression analyses exposing a poor-outcome patient group with a median survival of little more than two years [7]. Consequently, even after treatment, ACC patients face uncertain outcomes due to the likelihood for local recurrence, distant metastases, lack of targeted therapeutics, and intrinsic tumor diversity.

Hallmark chromosomal translocations in ACC tumors activate the *MYB* or related *MYBL1* genes, indicating that these are the most common drivers in ACC tumors [8,9,10]. The *NFIB* gene is a common translocation partner, creating t(6;9) and t(8;9) fusions for the *MYB* and *MYBL1* genes, respectively. However, less frequent translocations involving other genes also occur, suggesting that *NFIB* is not an obligatory fusion target [7]. Instead, the chromosomal translocations are thought to activate the expression of the *MYB* (or *MYBL1*) gene by recruiting distant enhancers that interact with the *MYB* promoter [11], implicating enhancer hijacking as a primary mechanism activating the *MYB* gene in ACC tumors. Thus, a thorough understanding of the promoter–enhancer interactions that occur in ACC tumors is essential for devising novel therapeutics that could disrupt these interactions.

Transcription of the *MYB* gene is tightly controlled and highly regulated throughout development in different tissues. The *MYB* promoter, upstream of exon 1, is G-C rich and responds to a variety of stimuli [12,13]. In some tissues, a secondary regulatory mechanism involving a transcriptional pause site in the first intron is also important [14,15,16,17]. For example, estrogen receptor-regulated RNA polymerase stalling controls *MYB* expression in some types of breast cancer [12]. In normal proliferating erythroid cells, this entire region, from the promoter through the length of the first intron, interacts with multiple distant enhancer elements forming a dynamic active chromatin hub [16]. Additionally, an alternative *MYB* promoter immediately upstream of the second exon has been implicated in the aberrant expression of *MYB* in some leukemia cell lines [18,19]. Aberrant alternative promoter activation was first implicated in oncogenesis at least 25 years ago [20] and evidence of its role in tumorigenesis has continued to increase [21]. However, the alternative *MYB* promoter has not previously been shown to play an important role in tumors or normal tissues.

Unique, tumor-specific interactions between a hijacked enhancer and the *MYB* gene promoter could provide a novel target for therapeutic intervention in ACC tumors. However, *MYB* is also highly overexpressed in ACC tumors that do not have detectable chromosomal translocations, and the mechanism of *MYB* activation in these tumors is unclear. We performed detailed investigations of the regulation of the *MYB* gene in ACC tumors. Surprisingly, we found that ACC tumors utilize a normally silent alternative promoter located in the first intron of the *MYB* gene. These results have important implications for devising possible strategies to disrupt Myb-driven oncogenesis that leads to ACC tumor formation.

## 2. Results

### 2.1. ACC Tumors Utilize Two MYB Gene Promoters

**Figure 1:**
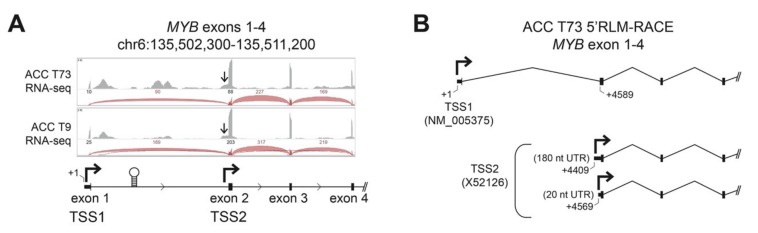
ACC tumors use two *MYB* gene promoters. (**A**) RNA-seq data from two ACC tumors is displayed in a genome browser view. Aligned RNA-seq read coverage is displayed as gray peaks above the gene track and the raw numbers of reads spanning splice junctions are indicated above the corresponding red arcs. The normal *MYB* gene promoter, TSS1, is upstream of exon 1. The first intron contains the RNA-polymerase attenuation site (stem-loop structure) and an alternative promoter (TSS2) upstream of exon 2 (second bent arrow). Arrows point at a shoulder of reads initiating upstream of exon 2, consistent with TSS2 use. (**B**) RNA Ligase-Mediated-Rapid Amplification of cDNA Ends (5′RLM-RACE) was performed on a frozen ACC tumor sample (T73) revealing transcripts from both TSS1 (designated +1 nt, RefSeq NM_005375) and TSS2 (GenBank X52126). Multiple transcription start sites were observed for TSS2, at positions +4409 and +4569, creating 180 and 20 nucleotide (nt) long 5′ Untranslated Regions (UTR), respectively.

Transcriptional regulation of the *MYB* gene has not been studied in detail in ACC tumors, but in most tissues and tumor types, transcription of the gene initiates upstream of exon 1 at the normal promoter, designated here as TSS1 (Transcription Start Site 1, Figure 1A) [12,13]. Detailed analyses of RNA-sequencing (RNA-seq) studies of ACC tumors [7,8] have revealed that nearly all ACC tumors have very few reads aligned to the first exon of the *MYB* gene, suggesting an anomaly in its transcriptional regulation in ACC. In addition to the normal TSS1 promoter, several additional regulatory elements have been described in the *MYB* gene. A regulatory RNA polymerase II pause site is located downstream of exon 1 in the first intron (Figure 1A, stem-loop structure), which binds several types of nuclear factors to control *MYB* gene expression in some cell types [12,22,23,24]. In addition, an infrequently used alternative promoter, designated here as TSS2, is located just upstream of exon 2 (Figure 1A) [18,19]. We visually inspected the RNA-seq reads from two frozen ACC tumors (T73 and T9; clinical information [7]). Figure 1A shows a genome browser view of the RNA-seq coverage of the first four exons of the *MYB* gene. We found markedly fewer reads aligned to exon 1 compared to exon 2 and the number of reads spliced from exon 1 to exon 2 was dramatically lower than those spliced from exon 2 to exon 3 (the raw number of reads is indicated above the arcs, which are displayed proportionally, Figure 1A). If transcription in these tumors began at TSS1 and continued through the remainder of the gene, the number of reads aligned to exon 1 and exon 2 should be approximately equal. Alternatively, if transcription began at TSS1 and the RNA polymerase stalled at the regulatory hairpin structure within intron 1, a buildup of reads upstream of the hairpin followed by many fewer reads on exon 2 would be expected. However, we observed many more reads on exon 2 than exon 1, which is most consistent with transcription skipping TSS1 and instead beginning at TSS2 (Figure 1A). We also observed reads upstream of exon 2 (arrows in Figure 1A) consistent with transcripts initiating from TSS2.

To confirm that ACC tumors used the TSS2 promoter, we mapped the 5′-ends of transcripts in ACC tumor T73 using RNA-Ligase Mediated-Rapid Amplification of cDNA Ends (5′RLM-RACE), followed by conventional ‘Sanger’ sequencing (Figure 1B). Although it is not a quantitative assay, 5′RLM-RACE specifically discovers the extreme 5′ ends of completely processed and capped mRNA transcripts and can be used to infer the promoter location and transcription start sites. In ACC tumor T73, we identified transcripts originating from the expected TSS1 promoter directly upstream of exon 1 (Figure 1B, RefSeq ID NM_005375). However, we also detected multiple transcripts that began in intron 1 directly upstream of exon 2 (Figure 1B, TSS2, GenBank X52126, Figure S1 and Table S1). These transcripts are unlikely to be read-through from TSS1 since RNA splicing would remove this intronic region. We conclude that these transcripts in ACC tumor T73 must originate from TSS2, as predicted from the sequencing data in Figure 1A. Detailed investigation of TSS2 transcripts in T73 revealed replicate transcripts from multiple potential transcription start sites that extended either ~180 or ~20 nucleotides (nt) upstream of exon 2 into intron 1 (Figure 1B, +4409 nt and +4569 nt, respectively). Only the ~20 nt TSS2 transcript has been previously described in the literature [19]. The ~180 nt TSS2 transcript appears to be novel.

Transcription initiating from TSS2 has been previously reported in some leukemia cell lines, where it accounted for a substantial proportion of total *MYB* transcripts [18,19]. However, TSS2 transcripts have not been described in human tumors or leukemia samples or in normal hematopoietic or epithelial cells and only one such transcript has been annotated (GenBank Accession X52126). Here, we provide the first evidence that *MYB* gene TSS2 is activated in an ACC tumor. 

### 2.2. Myb Transcription Factors Can Activate Cell-Type Specific MYB Promoters

**Figure 2:**
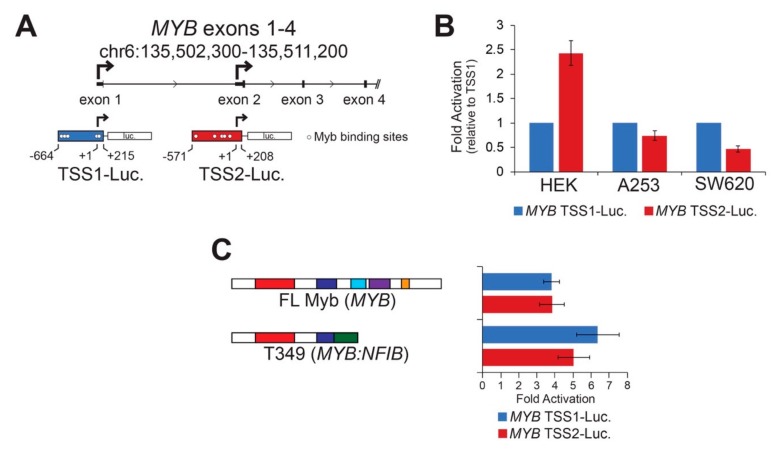
Cell type specific *MYB* promoters are activated by Myb transcription factors. (**A**) *MYB* promoter reporter constructs corresponding to TSS1 (blue) and TSS2 (red) are illustrated. Transcription start sites are designated +1 and indicated with bent arrows. Predicted high affinity (see Methods) Myb binding sites are indicated as open circles. (**B**) *MYB* promoters are activated differently in different cell types. The reporters above were transiently transfected into three cell types in the absence of exogenous activator. Fold activation was calculated relative to TSS1 (blue), which was set arbitrarily to a value of 1. (**C**) Both *MYB* promoters were activated by full-length and truncated Myb proteins. Full-length Myb (MYB) and a truncated Myb protein predicted from ACC tumor T349, which encodes *MYB* exons 1–8 fused to *NFIB* exons 11–12, were used in the reporter assay. This assay was performed in HEK293TN cells, but similar results were obtained in other cell lines. All experiments are displayed as an average of three biologically independent replicates, error bars represent the standard deviation.

*MYB* TSS2 has been described previously [18,19], but little is known about its regulation, function, or significance in human tumors. We utilized reporter assays to perform basic characterizations of both *MYB* promoters. *MYB* TSS1 and TSS2 were cloned upstream of the luciferase reporter gene (Figure 2A and Figure S2), then introduced into different cell lines where their basal activities were measured (Figure 2B). Both TSS1 and TSS2 functioned as bona fide promoters in these assays, but they displayed cell type-specific differences (Figure 2B). The two promoters displayed similar activities in A253 salivary gland epidermoid carcinoma cells, but TSS2 was more active than TSS1 in HEK293 human embryonic kidney cells, and the opposite was true in SW620 colorectal adenocarcinoma cells. These data confirm that the region upstream of exon 2 can act as a promoter in human cells and suggest that *MYB* promoter selection is regulated in a cell-type specific manner.

Sequence motif analyses [25] revealed known and novel transcription factor binding sites in each of the *MYB* promoters (Figure S2). Previously, we noted several transcription factor genes that were closely correlated to the levels of *MYB* including the homeobox protein EN1 and the SRY-related HMG protein SOX4 [7]. A more extensive analysis revealed additional transcription factors that were significantly more highly expressed in the *MYB*-expressing ACC tumors including the genes encoding homeobox proteins SIX3 and PBX1; additional SOX proteins SOX6, SOX8, SOX9, SOX10, and SOX11; the forkhead domain protein FOXO3B; the ETS family protein ETV6; and the important tumor suppressor protein TP53 [7]. Figure S2 shows the 500 nt immediately upstream of the TSS1 and TSS2 promoters, with the predicted binding sites for these families of transcription factors indicated. Note that these were only predicted from the sequence motifs and have not yet been confirmed through chromatin immunoprecipitation analyses.

The TSS1 promoter is GC-rich (71%) and has multiple binding sites for transcription factors like TP53, which has a GC-rich binding motif. However, DNA methylation at CpG sites does not appear to be important in regulating the TSS1 promoter in normal salivary glands or in ACC tumors [26]. The TSS2 promoter is only 37% GC and has numerous predicted binding sites for transcription factors that bind AT-rich motifs such as Foxo, Hox, SRY, and Myb proteins. The numerous predicted Myb binding sites in TSS2 suggest that once Myb becomes expressed in ACC tumors, a stimulatory loop may exist, allowing Myb to keep its own gene activated through the TSS2 promoter. Since gene expression changes elicited by Myb transcription factors are responsible for driving tumorigenesis in over 80% of ACC tumors [7,8], we investigated whether Myb could activate its own promoters. HEK293TN cells were transfected with each of the *MYB* promoter reporters along with concurrently expressed Myb proteins. Figure 2C shows the fold activation of the promoters by ectopically expressed Myb proteins relative to no Myb protein. We found both *MYB* promoters were activated similarly (4 to 7-fold) by full-length Myb and a Myb-Nfib fusion protein (T349, Figure 2C) [8]. The two *MYB* promoters were not significantly activated by a mutated Myb transcription factor harboring a point mutation that disrupts DNA-binding activity [27]. These data demonstrate that Myb transcription factors are capable of activating both *MYB* gene promoters, which could create stimulatory feedback loops in ACC tumors. Note that although TSS1 has no predicted MYB binding sites immediately upstream of the transcription start site, there are several predicted binding sites more than 500 nt away and just downstream of the start site. Myb activation of the TSS1 promoter could work through those sites or Myb could activate the promoter through an indirect mechanism.

### 2.3. TSS2 Transcripts Produce Myb Proteins with an N-terminal Truncation

**Figure 3:**
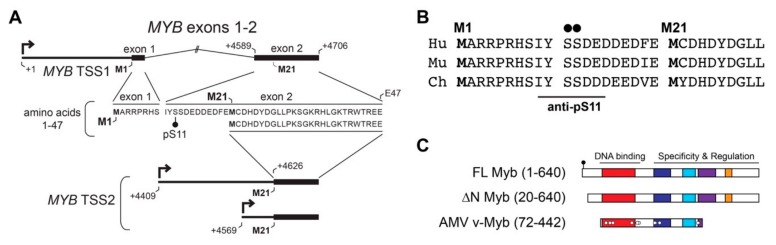
*MYB* TSS2 mRNA encodes an N-terminally truncated Myb protein isoform (∆N Myb). (**A**) *MYB* transcripts beginning at TSS1 (top) include exons 1 and 2, which encode amino acids 1–47 of the Myb protein (amino acid sequence below mRNA transcript). *MYB* TSS2 transcripts begin at +4409 nt or +4569 nt downstream of TSS1 (designed +1), skipping the entire first exon. These transcripts do not include the first start codon (M1) and are instead predicted to begin translation at residue M21, skipping the first 20 amino acids of the full-length Myb protein. (**B**) Conservation of the N-terminal domain of Myb encoded by TSS1 in human (Hu), murine (Mu), and chicken (Ch) proteins. (**C**) The full-length Myb protein (amino acids 1–640) encodes conserved DNA-binding and regulatory regions. TSS2 transcripts were predicted to encode a 20 amino acid N-terminal deletion, producing the ∆N Myb isoform (amino acids 20–640). The oncogenic Avian Myeloblastosis Virus (AMV) v-Myb (amino acids 72–442) also has a 72 amino acid N-terminal truncation.

To understand the importance of TSS2 use by ACC tumors, we investigated the effects on encoded Myb proteins. Figure 3A illustrates the mRNA isoforms encoded by TSS1 (top) and TSS2 (bottom) with the corresponding amino acid sequence derived from each (middle). Translation of full-length Myb begins at the first start codon located in exon 1 (M1) and together, exons 1 and 2 encode the first 47 amino acids (aa) of the full-length protein (Figure 3A). However, when TSS2 is utilized (*MYB* TSS2 lower mRNA, Figure 3A), transcripts begin within intron 1 and do not include the usual start codon (M1). Instead, translation is predicted to begin at the first in-frame start codon (M21) located in exon 2, thereby skipping the first 20 aa of the Myb protein. The N-terminal region of Myb proteins is highly conserved in evolution, and is nearly identical in the proteins from human, mouse, and chicken (Figure 3B), suggesting it plays an important role in Myb activity or regulation.

**Figure 4:**
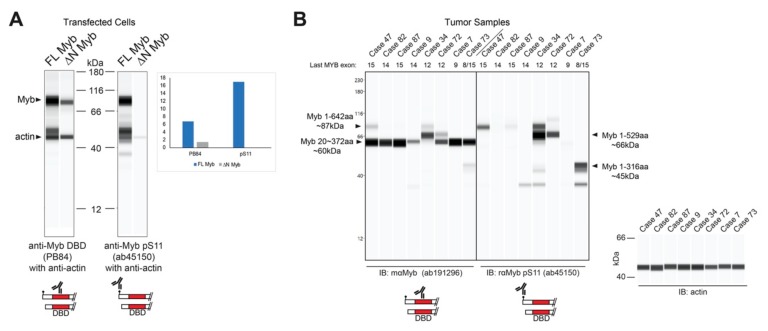
∆N Myb is not detected by a common Myb antibody. (**A**) Myb proteins were overexpressed in HEK293TN cells and lysates were probed with two anti-Myb antibodies and anti-actin using capillary blotting (see Materials and Methods). Rabbit serum that detects the DNA binding domain (PB84, left) detects both full-length (FL) and ∆N Myb isoforms. However, the popular anti-Myb pS11 antibody (ab45150, right) was unable to detect the ∆N Myb isoforms. Inset shows the quantification of FL Myb and ∆N Myb expression relative to the actin loading control for both anti-Myb antibodies. (**B**) Myb protein expression from frozen ACC tumors. Total lysates were probed with two anti-Myb antibodies and actin, then visualized using capillary blotting. An antibody that detects the DNA binding domain (ab191296; left) and the anti-Myb pS11 antibody (ab45150; right). Actin loading controls are shown at the right. The last transcribed exon of the MYB gene, estimated from RNA-seq data, is shown across the top. Note that the anti-pS11 antibody (right) only detects a fraction of the total Myb protein detected by the anti-DNA binding domain antibody (left). A second antiserum specific for the DNA binding domain gave similar results (see Figure S3).

The resulting Myb proteins, illustrated in Figure 3C, have several highly conserved domains comprising the DNA binding and regulatory regions of the protein. As described above, Myb proteins translated from TSS2 lack the first 20 amino acids, producing the ∆N Myb protein isoform (Figure 3C, ∆N Myb). Similarly, the oncogenic v-Myb protein encoded by Avian Myeloblastosis Virus (AMV) harbors a 72 amino acid N-terminal truncation (Figure 3C, v-Myb) [28]. The skipped N-terminal residues are highly conserved and contain a Casein kinase II phosphorylation site at serine residues 11 and 12 (pS11, pS12) (Figure 3A–C, black dots) [29]. The pS11 site serves as the epitope for a popular Myb antibody, which we exploited to illustrate full-length Myb and ∆N Myb protein isoform expression (Figure 4A). Engineered Myb proteins expressed from cDNA vectors in HEK293TN cells were subjected to Western blot analyses and probed with two Myb antibodies. An antiserum directed toward the DNA-binding domain (PB84, Myb DBD, [30]), which is an essential domain present in all Myb isoforms, detects both full-length and the ∆N Myb isoform. In contrast, the antibody directed toward the Myb pS11 residue (Myb pS11, ab45150) only detects full-length Myb, not ∆N Myb. This confirms that the ∆N Myb produced from TSS2 is not detected by the pS11 antiserum (ab45150), which is commonly used to detect Myb proteins in ACC samples.

Since the pS11 epitope is at the extreme N-terminal end, Myb proteins produced from TSS2 should be undetectable using an antibody like ab45150 that targets pS11. We analyzed the Myb protein expression in several *MYB*-expressing, frozen ACC tumor samples. As shown in Figure 4B, prominent Myb protein bands were detected with the anti-DBD antibody in all samples (left), but the anti-pS11 antibody only detected Myb proteins in some samples (right), suggesting that some ACC tumors harbored Myb proteins with N-terminal deletions that lack the pS11 epitope. This is consistent with the hypothesis that ACC tumors utilize the TSS2 promoter. However, the Myb proteins were consistently smaller than expected, suggesting that a C-terminal domain was also missing. From these results, we cannot distinguish whether the C-terminal truncated proteins accumulated in the tumors or were the result of partial proteolysis after the samples were collected. However, the smaller than expected Myb proteins were detected by several different antibodies (Figure S3), and changed size depending on the status of *MYB* gene translocations (e.g., case 34 in Figure 4B), indicating that they were not due to a non-specific background. Based on the molecular weights and epitope mapping, we estimate that these Myb proteins in ACC tumors extend to at least amino acid 372, or beyond *MYB* exon 8. Thus, at least some of the Myb proteins expressed by ACC tumors lack the N-terminus and the pS11 epitope/phosphorylation site, as predicted when the alternative TSS2 promoter is used.

The ∆N Myb protein isoform has intact DNA-binding and regulatory domains and is predicted to be a functional transcription factor. Similarly, the oncogenic v-Myb protein encoded by Avian Myeloblastosis Virus (AMV, Figure 3C) has an even larger 72 amino acid N-terminal truncation, yet retains its DNA-binding ability and is a functioning oncogenic transcription factor. We confirmed ∆N Myb was able to activate two known Myb regulated promoters [7] in reporter assays (Figure S4). Thus, the first 20 conserved amino acids of Myb including the phosphorylated residues, are not required for the DNA binding and transactivation activities of Myb transcription factors [31,32,33]. Still, the highly conserved nature of this region (Figure 3B) suggests it serves an important regulatory function.

### 2.4. ∆N Myb Encoded in ACC Tumors Has Unique Transcriptional Activity

**Figure 5:**
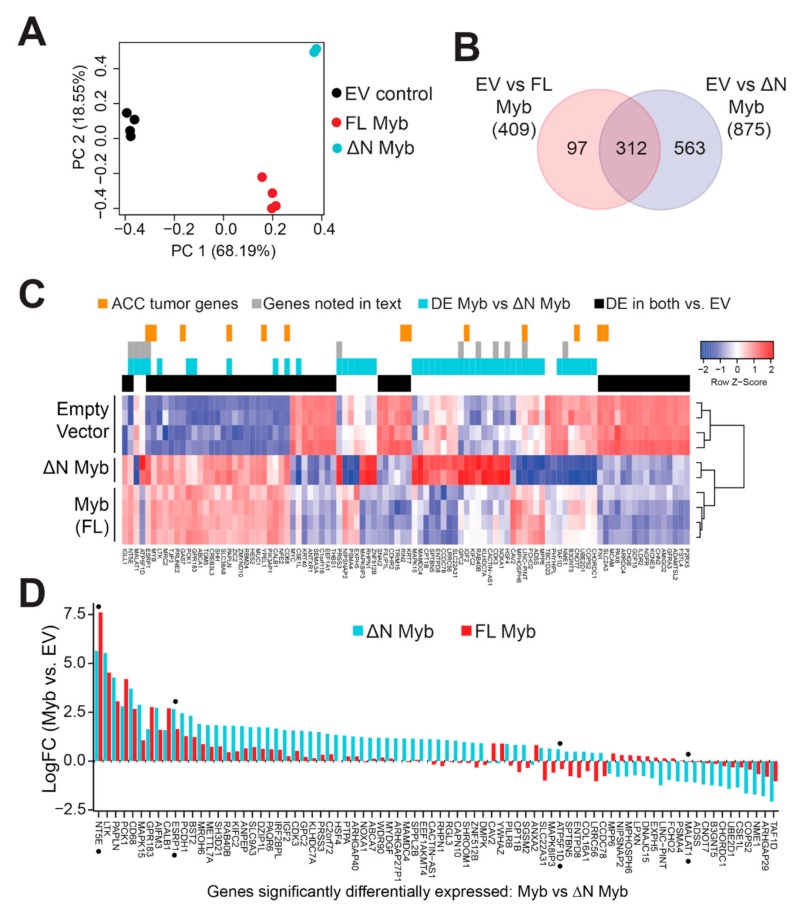
Myb and ∆N Myb transcription factors have overlapping, but distinct activities. (**A**) Myb proteins were ectopically expressed in SW620 cells using lentivirus vectors. Total RNA was harvested at 48 h post transduction and analyzed by RNA-sequencing. PCA analyses distinguished empty vector control (black) from full-length Myb (red) and ∆N Myb (cyan). (**B**) Myb isoforms regulated some overlapping, but also many uniquely regulated genes. Differentially expressed genes (2-fold change, BH corrected *p*-Value < 0.05) were identified for each Myb isoform versus empty vector control and the gene lists are compared in the Venn diagram. (**C**) A summary heatmap displays gene expression changes elicited by Myb transcription factors. The color keys above the heatmap indicate genes differentially expressed in Myb expressing ACC tumors versus Myb negative ACC tumors (orange), genes discussed in the text (gray), genes differentially expressed in Myb versus ∆N Myb (cyan), and genes differentially expressed by both Myb and ∆N Myb relative to empty vector control (black). (**D**) The bar chart displays the log fold change relative to empty vector of the 80 genes differentially regulated by Myb (red) versus ∆N Myb (cyan).

Minor changes in the Myb protein have been previously shown to have dramatic effects on its transcriptional activity and its ability to activate specific target genes [34,35,36,37]. We hypothesized that N-terminal truncation may alter the activity of Myb in ACC tumor cells. We used lentivirus vectors to express either full-length Myb or ∆N Myb and then used RNA-seq to measure subsequent changes in gene expression. We used SW620 colorectal adenocarcinoma cells, which express very low endogenous Myb expression, since both TSS1 and TSS2 were functional in those cells in our transfection assays (Figure 2B). We achieved up to ~95% transduction with moderate overexpression of Myb proteins, judged by western blot analysis (Figure S5). Principal component analysis (PCA) showed clear separation of the three treatments (Figure 5A). Full-length Myb (red) and ∆N Myb (cyan) were separated from empty vector control (EV, black) along the first component (PC1, horizontal axis), which explained almost 70% of the variation in the data. Even these initial analyses distinguished full-length Myb (red) from ∆N Myb (cyan) along the second component (PC2, vertical axis), which accounted for 18% of the variation (Figure 5A). The PCA plot highlighted an exciting difference in the gene expression signatures elicited by the two Myb protein isoforms, which differed only by 20 amino acids at their N-terminus.

We performed differential gene expression analyses comparing each of the Myb samples to the empty vector controls and identified hundreds of potential Myb regulated genes. Specifically, 409 genes were significantly up- or downregulated at least 2-fold by full-length Myb (adjusted *p*-Value < 0.05), and 875 genes were differentially regulated by ∆N Myb (Figure 5B, full list of DE genes Table S2). The Venn diagram (Figure 5B) compares these gene lists and shows that 312 genes were differentially regulated by both versions of Myb, but ∆N Myb caused an additional 563 genes to be up- or downregulated at least 2-fold. A heatmap summarizing the gene expression analyses is shown in Figure 5C (a larger version can be seen in Figure S6). Some of the differentially expressed genes are associated with important cell functions like cell cycle regulation (*CDK3*, *COPS2*, *HSF4*), while others have known oncogenic functions (*MALAT1*, *GPC2*, *LINC-PINT*) or are associated with metastases in a variety of tumors (*RAB40B*, *PRSS3*, *NME1*). Genes commonly regulated by Myb and ∆N Myb are indicated by the black color bar along the top of the heatmap; genes regulated differently by ∆N Myb and full-length Myb are indicated by the cyan color bar. Additionally, genes discussed later in this text are indicated in gray, and orange marks indicate genes previously found to be associated with Myb expression in ACC tumors [7] (Figure 5C).

The differences in activity between full-length and ∆N Myb were even more dramatic when the gene expression data are presented as a bar chart, which displays the genes differentially regulated by ∆N Myb (cyan) or full-length Myb (red; Figure 5D). Note that the scale in Figure 5D is log2 of fold-change, so 2.5 = 5.6-fold change. Some genes were regulated similarly by both Myb isoforms (e.g., at left), others were induced or repressed more dramatically by ∆N Myb or were regulated in completely opposite directions by ∆N Myb and full-length Myb proteins. In comparison to empty vector control, the *ESRP1* gene (denoted with black dot) is significantly activated by Myb ~3.1 fold (red bars) and ~6.4 fold by ∆N Myb (cyan bars; Figure 5D). Thus, *ESRP1* is activated ~2.0 fold more dramatically by ∆N Myb, which could be consistent with ∆N Myb being an “unleashed” version of Myb. However, the *NT5E* gene was activated 195-fold by full-length Myb, but only 50-fold by ∆N Myb (Figure 5D). We also observed genes that were regulated by one isoform but not the other, like the oncogenic lincRNA *MALAT1*, which is silenced by ∆N Myb, but is unchanged by full-length Myb. Finally, full-length Myb and ∆N Myb even regulated a few genes in opposite directions, for instance the *ATP5F1D* gene is silenced by full-length Myb, but activated by ∆N Myb relative to empty vector (Figure 5D). These data suggest that in some situations ∆N Myb may have a completely different transcriptional activity and specificity than its full-length counterpart.

### 2.5. ∆N Myb Uniquely Modulates Gene Sets Implicated in Neuronal Cell Migration

**Figure 6:**
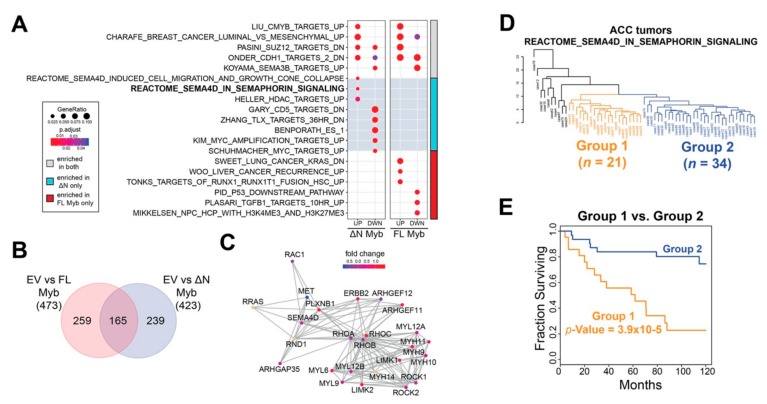
∆N Myb uniquely activates SEMA4D signaling, which is correlated with ACC patient survival. (**A**) Gene set enrichment analyses were performed using the significantly differentially expressed genes discovered in Figure 5. The top two gene sets significantly enriched for each category plus selected gene sets unique to each category are displayed. The color bar to the far right indicates the treatment in which the gene set was enriched in both Myb isoforms in gray, enrichment in only full-length Myb in red, and enrichment in only ∆N Myb in cyan. (**B**) Venn diagram of all the significantly enriched gene sets for each Myb isoform. (**C**) Gene network for the REACTOME_SEMA4D_IN_SEMAPHORIN_SIGNALING (R-HSA-400685) gene set. A total of 25 of the 32 genes are displayed, genes not included are *CD72*, *CDC42*, *LOC642076*, *MYL8P*, *MYL12A*, *PTPRC*, *RAC2*, *RHOG*, and *ROCKIP1*. Fold change in ∆N Myb treated SW620 cells versus empty vector control was used to color the gene nodes. (**D**) Unsupervised hierarchical clustering using the 22 genes from the REACTOME_SEMA4D_IN_SEMAPHORIN_SIGNALING gene set that were expressed in ACC tumors. Two major groups were defined using the resulting dendrogram, 21 tumors were in Group 1 (orange) and 34 tumors were in Group 2 (blue). The remainder of the ACC tumors clustered into multiple smaller groups and are colored black. (**E**) There was a significant difference in ACC patient survival when grouped based on SEMA4D signaling. Tumors were assigned to Group 1, Group 2, or excluded from these analyses based on the dendrogram in panel D.

To analyze differences in gene pathways, we used the ClusterProfiler R package to query the Molecular Signatures Database [38,39]. The results are summarized in Figure 6A, where the left panel displays gene sets discovered in ∆N Myb treated cells and the right panel displays gene sets discovered in Myb treated cells. The color bar to the far right indicates gene sets discovered in both Myb and ∆N Myb treated cells in gray, gene sets unique to ∆N Myb in cyan, and those unique to Myb in red (Figure 6A). Full enrichment results are provided in Table S3. Approximately 35% of the discovered gene sets (165/423 sets) were enriched in both full-length Myb and ∆N Myb expressing cells (Figure 6B), this included many of the top-ranking gene sets (categories denoted with gray in the side bar, Figure 6A) [40]. Furthermore, full-length Myb and ∆N Myb both activated genes that are overrepresented in a previously published c-Myb target gene list (LIU_CMYB_TARGETS_UP) [36] and silenced genes associated with *SEMA3B* expression (KOYAMA_SEMA3B_TARGETS_UP, Figure 6A) [41]. Thus, our enrichment results are consistent with previously published findings and our own findings that ∆N Myb and full-length Myb commonly regulated some genes.

However, gene expression analyses have also established that full-length Myb and ∆N Myb transcription factors regulated many genes differently (Figure 5B). We compared all the significantly up- and down-regulated genes associated with each Myb isoform and found that almost 70% of the enriched gene sets (239/423 sets) were unique to ∆N Myb (Figure 6B). A selected subset of these unique gene sets are included in Figure 6A (red and cyan side bar). Specifically, genes activated in cells that expressed ∆N Myb were significantly enriched in *SEMA4D* associated migratory cues (REACTOME_SEMA4D_INDUCED_CELL_MIGRATION_AND_GROWTH_CONE_COLLAPSE and REACTOME_SEMA4D_IN_SEMAPHORIN_SIGNALING [42,43]) (Figure 6A), while genes silenced in cells expressing ∆N Myb were significantly enriched in gene sets associated with immature or stem cell phenotypes (ZHANG_TLX_TARGETS_36HR_DN and BENPORATH_ES_1 [44,45]). Full-length Myb (categories indicated in red) upregulated genes enriched in *RUNX1* gene sets (TONKS_TARGETS_OF_RUNX1_RUNXT1_FUSION_HSC_UP [46]) and silenced genes were enriched in p53 targets (PID_P53_DOWNSTREAM_PATHWAY [47]) (Figure 6A).

SEMA4D signaling has long been associated with neural cell migration and is implicated in a number of tumors [48]. The REACTOME_SEMA4D_IN_SEMAPHORIN_SIGNALING gene set, or SEMA4D signaling for short includes 32 genes, six of which were significantly differentially expressed in ∆N Myb treated SW620 cells (*MYH9*, *PLXNB1*, *RAC2*, *ARHGEF11*, *RHOC*, and *LIMK2*). The SEMA4D signaling gene interaction network is displayed in Figure 6C and the gene nodes are colored according to the fold change in ∆N Myb treated SW620 cells versus empty vector control cells. Interactions between the Sema4D signaling molecule and its receptor Plexin-B1 have been implicated in the perineural invasion via chemo-attractive interactions in multiple tumors types [49]. This same pathway was studied in more mechanistic detail in breast carcinoma cells, where SEMA4D signaling via its receptor Plexin-B1 either activated or suppressed cell migration [50]. These contrasting effects were linked to intermediate signaling molecules: signaling through ErbB-2 stimulated migration, whereas signaling through Met suppressed migration. Indeed, in our RNA-seq experiments ∆N Myb upregulated expression of *SEMA4D*, *PLXNB1*, and *ERBB2* and downregulated *MET* (Figure 6C). Thus, the unique gene expression changes elicited by ∆N Myb in SW620 cells are consistent with SEMA4D stimulated cell migration.

### 2.6. Expression of ∆N Myb Is Linked to a Poor Outcome Subgroup of ACC Tumors

Previous studies have shown that Myb expression affects different genes in different cell types [35,36,51]. Although the RNA-seq results shown in Figure 5 came from the analysis of an epithelial cell line unrelated to ACC, the pathway analysis suggested that the SEMA4D signaling pathways were differentially affected. Using published RNA-seq data [7], we performed unsupervised hierarchical clustering of primary ACC tumors based on their expression of SEMA4D signaling pathway genes. The resulting dendrogram is displayed in Figure 6D. ACC tumors sorted into distinct groups, indicating that the SEMA4D signaling gene set captured meaningful biological variation in these tumors. The majority of ACC tumors were sorted into two large groups with 21 tumors in Group 1 (orange) and 34 tumors in Group 2 (blue; Figure 6D). The remainder of the ACC tumors were sorted into many small groups (colored in black to the left side of the dendrogram Figure 6D) and excluded from further analyses due to the small size of these groups (10 individuals in six groups total). Kaplan–Meier analysis was used to evaluate the prognoses of the two main groups of ACC tumors (Figure 6E). When the ACC tumors were divided according to the dendrogram in Figure 6D, there was a significant difference in patient survival. Group 2 (blue) was on par with the average survival for ACC patients, with a 5-year survival rate above 80% (blue, Figure 6E). In contrast, Group 1 (orange) had significantly poorer survival (log-rank *p*-Value = 3.9 × 10^−5^), with a median survival of 61.7 months and a much lower 5-year survival of ~55% (orange, Figure 6E). Thus, although we identified the SEMA4D pathway by analyzing the effects of Myb expression on a different cell type, activation of the SEMA4D signaling pathway genes were significantly associated with differential survival of ACC patients.

We conclude that the Myb proteins with a 20 amino acid N-terminal deletion, as expressed in ACC tumors through the use of alternative promoter TSS2, have unique transcriptional activities, allowing ∆N Myb to activate and silence many genes that full-length Myb did not, including important genes involved in cell migration and perineural invasion that may correlate with worse overall survival.

## 3. Discussion

Here, we provide the first evidence that ACC tumors used an alternative *MYB* promoter, leading to the expression of N-terminally truncated Myb proteins. We demonstrated that *MYB* promoters have cell-type specific activity and *MYB* TSS2 activation occurs in ACC tumors, suggesting that the underlying cause of TSS2 activation could be specific to ACC tumors. Chromosomal translocations are an important feature of ACC tumors and have many established roles in activating the *MYB* gene, one of which is to recruit enhancers downstream of the *NFIB* gene to interact with *MYB* TSS1, stimulating its expression [11]. It is probable that *MYB* TSS2 interacts with hijacked enhancers in ACC tumors that have chromosomal translocations, although this remains to be tested. This could be an important observation for developing therapies that target promoter–enhancer interactions.

The implications of promoter selection extend far beyond transcriptional regulation. Studies have shown alternative promoter use provides a mechanism to modulate protein expression and activity, where for instance, N-terminally truncated proteins produced from alternative promoters can have distinct functional activities. We hypothesized that ∆N Myb may have altered activity in ACC tumors and thus used RNA-seq to investigate the transcriptional activity of the Myb and ∆N Myb protein isoforms. We found that these transcription factor isoforms, which have identical DNA-binding domains, regulated hundreds of genes differently. Although the ∆N Myb lacks only 20 amino acids at the N-terminus, it had vastly different activity in the cells we used, where it not only activated different primary targets, but also different downstream pathways altogether. The N-terminal region of Myb is highly conserved, indicating an important function. Early studies implicated N-terminal truncation in oncogenesis, a mere 20 amino acid N-terminal truncation was sufficient to induce rapid-onset tumors when expressed in chickens [52]. While, phosphorylation of the pS11 and pS12 residues increased the specificity of full-length Myb by destabilizing DNA-binding [33,53], this effect was overcome by protein-to-protein interactions with co-factors that anchored Myb to DNA [33]. It is clear that N-terminally truncated Myb proteins are capable of binding and activating transcription of target genes. Thus, the mechanism responsible for the observed differences in Myb isoform activity is not clear.

From our RNA-seq analyses, a picture emerged potentially implicating ∆N Myb in ACC tumor migration and perineural invasion. In both Myb and ∆N Myb expressing cells, the largely chemo-repulsive, anti-tumorigenic SEMA3B associated migratory cues were downregulated [48]. In addition, ∆N Myb expressing cells alone displayed activated SEMA4D chemo-attractive, pro-tumorigenic migratory cues. Most ACC tumors express *SEMA4D*, and its receptor PlexinB1 is upregulated 5-fold in ACC tumors compared to normal salivary gland [8], findings consistent with activated SEMA4D signaling. We found that classifying ACC tumors according to SEMA4D signaling identified a significantly poorer outcome subgroup of tumors. SEMA4D signaling has been implicated in the migration and invasiveness of a variety of tumors. In epithelial cells, SEMA4D triggered invasive growth [54] and stimulated migration in breast cancer cells in concert with ErbB-2 signaling [50]. Overexpression of the SEMA4D receptor, PlexinB1, was correlated with invasiveness and metastasis in prostate tumors [55]. Finally, SEMA4D signaling is implicated in perineural invasion, a hallmark of ACC tumors [49]. Thus, our results potentially link three disjointed aspects of ACC tumors; expression of a previously unreported ∆N Myb isoform activated SEMA4D signaling, which is in turn implicated in perineural invasion and patient outcome.

We conclude that ACC tumors utilize a rare alternative *MYB* promoter. Our RNA-seq analyses revealed that ∆N Myb can differently activate or silence hundreds of genes, indicating that N-terminal truncation via *MYB* TSS2 activation qualitatively altered the specificity of Myb transcription factors. ∆N Myb alone was able to silence anti-tumorigenic neuronal migratory signals while also stimulating pro-tumorigenic neuronal migratory cues. Finally, expression of these same pro-tumorigenic neuronal migratory cues in ACC tumors identified a significantly poorer outcome subgroup of ACC tumors. These results potentially implicate ∆N Myb in stimulating perineural invasion in ACC tumors, the mechanisms of which are still largely unknown [56]. It will be exciting to see future studies that fully elucidate the role of TSS2 in *MYB* gene activation and expression, the extent of its interaction with hijacked enhancers, and its full functional consequences in ACC tumors.

## 4. Materials and Methods

### 4.1. Cell Culture and Luciferase Assays

Human Kidney 293TN Producer cells (HEK239TN; System Biosciences, 2438 Embarcadero Way, Palo Alto, CA 94303 USA) and SW620 colorectal carcinoma cells (ATCC, American Type Culture Collection, 10801 University Boulevard, Manassas, VA 20110 USA; CCL-227) were cultured in Dulbecco’s Modified Eagle’s Medium (DMEM; ATCC) supplemented with 5% (*v*/*v*) fetal bovine serum, 5% (*v*/*v*) newborn calf serum (Rocky Mountain Biologicals, Inc., 6015 Greg’s Way, Missoula, MT, USA), and 1% antibiotic-antimycotic. A-253 epidermoid carcinoma cells (ATCC; HTB-41) were cultured in McCoy’s 5A medium, supplemented with 5% (*v*/*v*) fetal bovine serum, 5% (*v*/*v*) newborn calf serum, and 1% antibiotic-antimycotic. All cells were cultured at 37°C in 5% CO_2_. Media and supplements were purchased from ThermoFisher (168 Third Avenue, Waltham, MA USA 02451) unless otherwise indicated. SW620 cells used for RNA-seq analyses were validated using the ATCC cell line authentication service in August 2019.

For reporter assays, cells were seeded in 24 well plates with approximately 4–6 × 10^4^ cells per well. After 24 h of growth, cells were transiently co-transfected with 50 ng of luciferase reporter plasmid plus 50 ng of activator plasmid (*MYB* cDNAs cloned into pcDNA3.0), or just reporter plasmid without activator. Transfections were performed in duplicate using the TransIT-2020 transfection reagent (Mirus Bio LLC, 545 Science Dr., Madison, WI 53711 USA), according to the manufacturer’s instructions. Cells were harvested, and firefly luciferase activity was measured after 48 h using the Luciferase Assay System (Promega, 2800 Woods Hollow Road, Madison, WI 53711). Background subtracted data were normalized as stated in the Results Section. Reporter gene data are an average of three independent biological replicates, with error bars representing the standard deviation.

### 4.2. Tumor RNA-Seq

ACC RNA-seq data processing and analysis were performed as previously [7], and data were downloaded from the NCBI BioProject database using study accession number PRJNA287156. Specifically, de-identified salivary gland adenoid cystic carcinoma FFPE and frozen tumor samples were obtained from the Salivary Gland Tumor Biorepository (MD Anderson Cancer Center, Houston, TX, USA). All samples were collected with informed consent of the donors, and studies were conducted in accordance with the principle of the Declaration of Helsinki. All studies were performed with Institutional Review Board-approved protocols. Exon counts were tabulated as the number of normalized RNA-seq reads that mapped to each *MYB* exon. RNA-seq from two frozen ACC tumors (T9 and T73) have been deposited in the NCBI BioProject (accession PRJNA573669).

### 4.3. SW620 RNA-Seq

SW620 cells were infected with concentrated lentiviral particles, so that 70–90% of cells were Green Fluorescent Protein (GFP) positive. RNA and total protein were harvested 48 h post transduction and submitted to western blotting and RNA-seq. RNA-seq experiments were performed as biological replicates. First, empty vector and Myb elicited gene expression was measured, then a second experiment including empty vector, Myb, and ∆N Myb was performed. Total RNA was extracted from cell pellets using the RNeasy Plus Mini Kit (Qiagen, 19300 Germantown Road, Germantown, MD 20874). Ribosomal RNA was removed with the RiboGone Kit (Takara Bio, 1290 Terra Bella Ave., Mountain View, CA 94043, USA), followed by cDNA synthesis using the SMARTer Universal Low Input RNA Kit for Sequencing (Takara Bio). Libraries were prepared using the Ion Plus Fragment Library Kit (ThermoFisher, 168 Third Avenue, Waltham, MA USA 02451) and sequenced using the Ion S5 Systems (ThermoFisher) in the Analytical and Translational Genomics Shared Resource at the University of New Mexico Comprehensive Cancer Center. Resulting RNA-seq reads were aligned to the human genome (GRCh37; hg19) using TMAP (v5.2.25) and gene counts were calculated using HT-Seq. Data were analyzed using R v3.5.1 using the edgeR (v3.24.0), DESeq (v1.22.1), ggplot2 (v3.1.0), RUVSeq (v1.16.0), limma (v3.38.2), mSIGDB (v6.1.1), survival (v3.6.1), stats (v3.6.1), and clusterProfiler (v3.10.1) packages [39,57,58,59,60]. RNA-seq data are available for download from NCBI BioProject using accession number PRJNA573669.

### 4.4. 5′RLM-RACE

Total RNA was extracted from frozen ACC tumors using the RNeasy total RNA extraction kit (Qiagen), according to the manufacturer’s specifications. 5′RLM-RACE was performed using the Generacer RLM RACE Kit (ThermoFisher), according to the manufacturer’s instructions. Briefly, total mRNA was dephosphorylated, de-capped, then the Generacer oligo ligated to the 5′ends. Reverse transcription was performed using either the provided oligo-dT primer or a gene specific primer to exon 8. A nested PCR reaction was used to amplify the products (Table S1). A mixture of PCR products were TOPO cloned (ThermoFisher) and Sanger sequenced to verify the insert sequence (Table S1).

### 4.5. Protein and Western Blots

Cell pellets were lysed in RIPA Lysis and Extraction Buffer (ThermoFisher, #89900) with protease inhibitor for 10 min on ice followed by sonication (Bioruptor, high, 30 s on/30 s off, 5–10 min, Diagenode Inc., 400 Morris Avenue, Suite 101, Denville, NJ 07834, USA). Capillary Western blotting was performed using the WES automated system (ProteinSimple, 3001 Orchard Parkway, San Jose, CA 95134 USA), all results were verified via traditional blotting methods. Cell lysates were diluted with a sample of 0.1× Sample Buffer (ProteinSimple) to a concentration of ~0.6 ug/uL. Protein separation and quantification was performed using the 12–230 kDa ladder, according to the manufacturer’s instructions. Antibodies used were as follows: rabbit anti-Myb (Rabbit antisera PB84, directed against amino acids 72–192 of Myb protein, 1:100, [30]), rabbit anti-Myb pS11 (ab45150; 1:200, Abcam, 1 Kendall Square, Suite B2304 Cambridge, MA 02139-1517, USA), and anti-βactin (1:100). Ready to use mouse (042-05) and rabbit (042-206) HRP-conjugated secondary antibodies were purchased from ProteinSimple. Myb antibodies were validated using Myb protein expressed from plasmid cDNA (positive control) and paired untransfected HEK293TN cell lysate (negative control), which does not express Myb.

### 4.6. Cloning

All MYB expression vectors were cloned into pcDNA3.0 as previously described [8]. Ectopically expressed Myb encoded the full-length proteins, ∆N Myb has a 20 amino acid N-terminal truncation described in the text. T349 is a C-terminally truncated Myb protein predicted from an ACC tumor, it includes MYB exons 1–8 fused to NFIB exons 11–12 [8]. The uc022bdo UCSC transcript was used as the *NFIB* gene reference, exons 11–12 encodes 73 amino acids. Reporter plasmids were cloned as follows: a 879 bp fragment of MYB TSS1 was amplified from genomic DNA using MYB TSS1 FW and RV primers (Table S1) and inserted into pGL3-basic with NheI and HindIII restriction sites. A 779 bp fragment of MYB TSS2 was amplified from genomic DNA using MYB TSS2 FW and RV primers (Table S1) and inserted into pGL3-basic with NheI and XhoI restriction sites. Reporter plasmid inserts were sequence verified via Sanger sequencing (Table S1).

### 4.7. Promoter Motif Analyses

Transcription factor binding motifs were discovered using the transcription factor affinity prediction (TRAP) set of web tools [25]. High quality motifs were defined as having a weight score above 2.5.

## 5. Conclusions

These studies were derived from our detailed RNA-seq analysis of archived ACC tumor samples [7,8], which led us to the finding that ACC tumors utilize an alternative promoter for the *MYB* gene, the major oncogenic ‘driver’ gene in these tumors. Alternative promoter use is not uncommon in human genes, but activation of the *MYB* gene in ACC tumors is thought to occur through chromosomal translocations that lead to ‘enhancer hijacking’ when distant enhancers are brought to the vicinity of the MYB gene, allowing its aberrant expression in ACC tumor cells [11]. Thus, it might be possible to design therapeutics to disrupt the tumor-specific promoter–enhancer interactions that occur in ACC tumors. It became important to analyze the *MYB* promoter, to ensure the correct promoter–enhancer interactions were targeted.

Our striking results showed that ACC tumors utilize an alternative *MYB* promoter that is not usually used in normal cells or other *MYB*-positive tumors. As a consequence, the ACC tumor cells express Myb proteins with an unexpected N-terminal deletion, which removes a highly conserved 20 amino acid segment of the protein. This deletion does not affect the ability of the ∆N Myb to activate reporter genes, but it changes the spectra of genes activated when the protein is expressed in cells, leading to altered expression of hundreds of genes. Thus, a very small change in Myb protein can have a very large impact on the activity of the oncoprotein, perhaps helping to explain why the Myb proteins expressed from the alternative promoter are the driver oncogenes in ACC tumors.

## Supplementary Materials

The following are available online at https://www.mdpi.com/2072-6694/12/1/45/s1, Figure S1: Details of 5′ RLM-RACE MYB TSS2 Products, Figure S2: Predicted transcription factor binding sites for the *MYB* promoter reporters., Figure S3: Myb proteins in ACC tumors detected by Western blotting, Figure S4: ∆N Myb proteins have similar activities to full-length Myb in reporter assays, Figure S5: Western blot of ectopically expressed Myb isoforms, Figure S6: Large heatmap of SW620 RNA-seq, Table S1: PCR primers and Sanger sequencing, Table S2: SW620 cells expressing RNA-seq data, Table S3: Over-representation analysis results.

## References

[1] DeAngelis A.F., Tsui A., Wiesenfeld D., Chandu A. (2011). Outcomes of patients with adenoid cystic carcinoma of the minor salivary glands. Int. J. Oral Maxillofac. Surg..

[2] Jones A.S., Hamilton J.W., Rowley H., Husband D., Helliwell T.R. (1997). Adenoid cystic carcinoma of the head and neck. Clin. Otolaryngol. Allied Sci..

[3] Spiro R.H. (1997). Distant metastasis in adenoid cystic carcinoma of salivary origin. Am. J. Surg..

[4] Van der Wal J.E., Becking A.G., Snow G.B., van der Waal I. (2002). Distant metastases of adenoid cystic carcinoma of the salivary glands and the value of diagnostic examinations during follow-up. Head Neck.

[5] Gil Z., Carlson D.L., Gupta A., Lee N., Hoppe B., Shah J.P., Kraus D.H. (2009). Patterns and incidence of neural invasion in patients with cancers of the paranasal sinuses. Arch. Otolaryngol. Head Neck Surg..

[6] Dantas A.N., Morais E.F., Macedo R.A., Tinoco J.M., Morais Mde L. (2015). Clinicopathological characteristics and perineural invasion in adenoid cystic carcinoma: A systematic review. Braz. J. Otorhinolaryngol..

[7] Frerich C.A., Brayer K.J., Painter B.M., Kang H., Mitani Y., El-Naggar A., Ness S.A. (2018). Transcriptomes define distinct subgroups of salivary gland adenoid cystic carcinoma with different driver mutations and outcomes. Oncotarget.

[8] Brayer K.J., Frerich C.A., Kang H., Ness S.A. (2016). Recurrent fusions in MYB and MYBL1 define a common, transcription factor-driven oncogenic pathway in salivary gland adenoid cystic carcinoma. Cancer Discov..

[9] Mitani Y., Liu B., Rao P.H., Borra V.J., Zafereo M., Weber R.S., Kies M., Lozano G., Futreal P.A., Caulin C. (2016). Novel MYBL1 gene rearrangements with recurrent MYBL1-NFIB fusions in salivary adenoid cystic carcinomas lacking t(6;9) translocations. Clin. Cancer Res..

[10] Persson M., Andren Y., Mark J., Horlings H.M., Persson F., Stenman G. (2009). Recurrent fusion of MYB and NFIB transcription factor genes in carcinomas of the breast and head and neck. Proc. Natl. Acad. Sci. USA.

[11] Drier Y., Cotton M.J., Williamson K.E., Gillespie S.M., Ryan R.J., Kluk M.J., Carey C.D., Rodig S.J., Sholl L.M., Afrogheh A.H. (2016). An oncogenic MYB feedback loop drives alternate cell fates in adenoid cystic carcinoma. Nat. Genet..

[12] Drabsch Y., Hugo H., Zhang R., Dowhan D., Miao Y., Gewirtz A., Barry S., Ramsay R., Gonda T. (2007). Mechanism of and requirement for estrogen-regulated MYB expression in estrogen-receptor-positive breast cancer cells. Proc. Natl. Acad. Sci. USA.

[13] Lauder A., Castellanos A., Weston K. (2001). c-Myb transcription is activated by protein kinase B (PKB) following interleukin 2 stimulation of Tcells and is required for PKB-mediated protection from apoptosis. Mol. Cell. Biol..

[14] Hugo H., Cures A., Suraweera N., Drabsch Y., Purcell D., Mantamadiotis T., Phillips W., Dobrovic A., Zupi G., Gonda T.J. (2006). Mutations in the MYB intron I regulatory sequence increase transcription in colon cancers. Genes Chromosomes Cancer.

[15] Pereira L.A., Hugo H.J., Malaterre J., Huiling X., Sonza S., Cures A., Purcell D.F., Ramsland P.A., Gerondakis S., Gonda T.J. (2015). MYB elongation is regulated by the nucleic acid binding of NFkappaB p50 to the intronic stem-loop region. PLoS ONE.

[16] Stadhouders R., Thongjuea S., Andrieu-Soler C., Palstra R.J., Bryne J.C., van den Heuvel A., Stevens M., de Boer E., Kockx C., van der Sloot A. (2012). Dynamic long-range chromatin interactions control Myb proto-oncogene transcription during erythroid development. EMBO J..

[17] Yuan W. (2000). Intron 1 rather than 5′ flanking sequence mediates cell type-specific expression of c-myb at level of transcription elongation. Biochim. Biophys. Acta.

[18] Dasse E., Volpe G., Walton D.S., Wilson N., Del Pozzo W., O’Neill L.P., Slany R.K., Frampton J., Dumon S. (2012). Distinct regulation of c-myb gene expression by HoxA9, Meis1 and Pbx proteins in normal hematopoietic progenitors and transformed myeloid cells. Blood Cancer J..

[19] Jacobs S.M., Gorse K.M., Westin E.H. (1994). Identification of a second promoter in the human c-myb proto-oncogene. Oncogene.

[20] Marcu K.B., Bossone S.A., Patel A.J. (1992). myc function and regulation. Annu. Rev. Biochem..

[21] Davuluri R.V., Suzuki Y., Sugano S., Plass C., Huang T.H. (2008). The functional consequences of alternative promoter use in mammalian genomes. Trends Genet..

[22] Manzella L., Gualdi R., Perrotti D., Nicolaides N.C., Girlando G., Giuffrida M.A., Messina A., Calabretta B. (2000). The interferon regulatory factors 1 and 2 bind to a segment of the human c-myb first intron: Possible role in the regulation of c-myb expression. Exp. Cell Res..

[23] Reddy D.C., Reddy E.P. (1989). Differential binding of nuclear factors to the intron 1 sequences containing the transcriptional pause site correlates with c-myb expresion. Proc. Natl. Acad. Sci. USA.

[24] Watson R.J. (1988). A transcriptional arrest mechanism involved in controlling constitutive levels of mouse c-myb mRNA. Oncogene.

[25] Thomas-Chollier M., Hufton A., Heinig M., O’Keeffe S., Masri N.E., Roider H.G., Manke T., Vingron M. (2011). Transcription factor binding predictions using TRAP for the analysis of ChIP-seq data and regulatory SNPs. Nat. Protoc..

[26] Shao C., Bai W., Junn J.C., Uemura M., Hennessey P.T., Zaboli D., Sidransky D., Califano J.A., Ha P.K. (2011). Evaluation of MYB promoter methylation in salivary adenoid cystic carcinoma. Oral Oncol..

[27] Frampton J., Gibson T.J., Ness S.A., Döderlein G., Graf T. (1991). Proposed structure for the DNA-binding domain of the Myb oncoprotein based on model building and mutational analysis. Protein Eng..

[28] George O.L., Ness S.A. (2014). Situational awareness: Regulation of the myb transcription factor in differentiation, the cell cycle and oncogenesis. Cancers.

[29] Cures A., House C., Kanei-Ishii C., Kemp B., Ramsay R.G. (2001). Constitutive c-Myb amino-terminal phosphorylation and DNA binding activity uncoupled during entry and passage through the cell cycle. Oncogene.

[30] Dash A.B., Orrico F.C., Ness S.A. (1996). The EVES motif mediates both intermolecular and intramolecular regulation of c-Myb. Genes Dev..

[31] Dini P.W., Lipsick J.S. (1993). Oncogenic truncation of the first repeat of c-Myb decreases DNA binding in vitro and in vivo. Mol. Cell. Biol..

[32] Oelgeschläger M., Kowenz-Leutz E., Schreek S., Leutz A., Lüscher B. (2001). Tumorigenic N-terminal deletions of c-Myb modulate DNA binding, transactivation and cooperativity with C/EBP. Oncogene.

[33] Oelgeschläger M., Krieg J., Lüscher-Firzlaff J.M., Lüscher B. (1995). Casein kinase II phosphorylation site mutations in c-Myb affect DNA binding and transcriptional cooperativity with NF-M. Mol. Cell. Biol..

[34] Introna M., Golay J., Frampton J., Nakano T., Ness S., Graf T. (1990). Mutations in v-myb alter the differentiation of myelomonocytic cells transformed by the oncogene. Cell.

[35] Lei W., Rushton J.J., Davis L.M., Liu F., Ness S.A. (2004). Positive and negative determinants of target gene specificity in Myb transcription factors. J. Biol. Chem..

[36] Liu F., Lei W., O’Rourke J.P., Ness S.A. (2006). Oncogenic mutations cause dramatic, qualitative changes in the transcriptional activity of c-Myb. Oncogene.

[37] Ness S.A., Kowenz-Leutz E., Casini T., Graf T., Leutz A. (1993). Myb and NF-M: Combinatorial activators of myeloid genes in heterologous cell types. Genes Dev..

[38] Subramanian A., Tamayo P., Mootha V.K., Mukherjee S., Ebert B.L., Gillette M.A., Paulovich A., Pomeroy S.L., Golub T.R., Lander E.S. (2005). Gene set enrichment analysis: A knowledge-based approach for interpreting genome-wide expression profiles. Proc. Natl. Acad. Sci. USA.

[39] Yu G., Wang L.G., Han Y., He Q.Y. (2012). clusterProfiler: An R package for comparing biological themes among gene clusters. OMICS.

[40] Pinero J., Queralt-Rosinach N., Bravo A., Deu-Pons J., Bauer-Mehren A., Baron M., Sanz F., Furlong L.I. (2015). DisGeNET: A discovery platform for the dynamical exploration of human diseases and their genes. Database.

[41] Koyama N., Zhang J., Miyazawa H., Tanaka T., Su X., Hagiwara K. (2008). Identification of IGFBP-6 as an effector of the tumor suppressor activity of SEMA3B. Oncogene.

[42] Garapati P. Sema4D Induced Cell Migration and Growth-Cone Collapse. https://www.reactome.org/content/detail/R-HSA-416572.

[43] Garapati P. Sema4D in Semaphorin Signaling. https://www.reactome.org/content/detail/R-HSA-400685.

[44] Ben-Porath I., Thomson M.W., Carey V.J., Ge R., Bell G.W., Regev A., Weinberg R.A. (2008). An embryonic stem cell-like gene expression signature in poorly differentiated aggressive human tumors. Nat. Genet..

[45] Zhang C.L., Zou Y., He W., Gage F.H., Evans R.M. (2008). A role for adult TLX-positive neural stem cells in learning and behaviour. Nature.

[46] Tonks A., Pearn L., Musson M., Gilkes A., Mills K.I., Burnett A.K., Darley R.L. (2007). Transcriptional dysregulation mediated by RUNX1-RUNX1T1 in normal human progenitor cells and in acute myeloid leukaemia. Leukemia.

[47] Schaefer C.F., Anthony K., Krupa S., Buchoff J., Day M., Hannay T., Buetow K.H. (2009). PID: The pathway interaction database. Nucleic Acids Res..

[48] Capparuccia L., Tamagnone L. (2009). Semaphorin signaling in cancer cells and in cells of the tumor microenvironment-two sides of a coin. J. Cell Sci..

[49] Binmadi N.O., Yang Y.H., Zhou H., Proia P., Lin Y.L., De Paula A.M., Guimaraes A.L., Poswar F.O., Sundararajan D., Basile J.R. (2012). Plexin-B1 and semaphorin 4D cooperate to promote perineural invasion in a RhoA/ROK-dependent manner. Am. J. Pathol..

[50] Swiercz J.M., Worzfeld T., Offermanns S. (2008). ErbB-2 and met reciprocally regulate cellular signaling via plexin-B1. J. Biol. Chem..

[51] Rushton J.J., Davis L.M., Lei W., Mo X., Leutz A., Ness S.A. (2003). Distinct changes in gene expression induced by A-Myb, B-Myb and c-Myb proteins. Oncogene.

[52] Jiang W., Kanter M.R., Dunkel I., Ramsay R.G., Beemon K.L., Hayward W.S. (1997). Minimal truncation of the c-myb gene product in rapid-onset B-cell lymphoma. J. Virol..

[53] Ramsay R.G., Ishii S., Gonda T.J. (1991). Increase in specific DNA binding by carboxyl truncation suggests a mechanism for activation of Myb. Oncogene.

[54] Giordano S., Corso S., Conrotto P., Artigiani S., Gilestro G., Barberis D., Tamagnone L., Comoglio P.M. (2002). The semaphorin 4D receptor controls invasive growth by coupling with Met. Nat. Cell Biol..

[55] Wong O.G., Nitkunan T., Oinuma I., Zhou C., Blanc V., Brown R.S., Bott S.R., Nariculam J., Box G., Munson P. (2007). Plexin-B1 mutations in prostate cancer. Proc. Natl. Acad. Sci. USA.

[56] Bakst R.L., Glastonbury C.M., Parvathaneni U., Katabi N., Hu K.S., Yom S.S. (2019). Perineural Invasion and Perineural Tumor Spread in Head and Neck Cancer. Int. J. Radiat. Oncol. Biol. Phys..

[57] Anders S., Huber W. (2010). Differential expression analysis for sequence count data. Genome Biol..

[58] Risso D., Ngai J., Speed T.P., Dudoit S. (2014). Normalization of RNA-seq data using factor analysis of control genes or samples. Nat. Biotechnol..

[59] Robinson M.D., McCarthy D.J., Smyth G.K. (2010). edgeR: A Bioconductor package for differential expression analysis of digital gene expression data. Bioinformatics.

[60] Wickham H. ggplot2-Elegant Graphics for Data Analysis. https://www.springer.com/gp/book/9780387981413.

