# Supplementary material for: N-Terminal Truncated Myb with New Transcriptional Activity Produced Through Use of an Alternative MYB Promoter in Salivary Gland Adenoid Cystic Carcinoma
Source: Cancers (Basel). 2019 Dec 21;12(1):45. doi: 10.3390/cancers12010045 (PMC7016764; doi:10.3390/cancers12010045)
Supplement: Supplementary file 1 [file cancers-12-00045-s001.zip › supplementary figures.pdf]

Figure S1

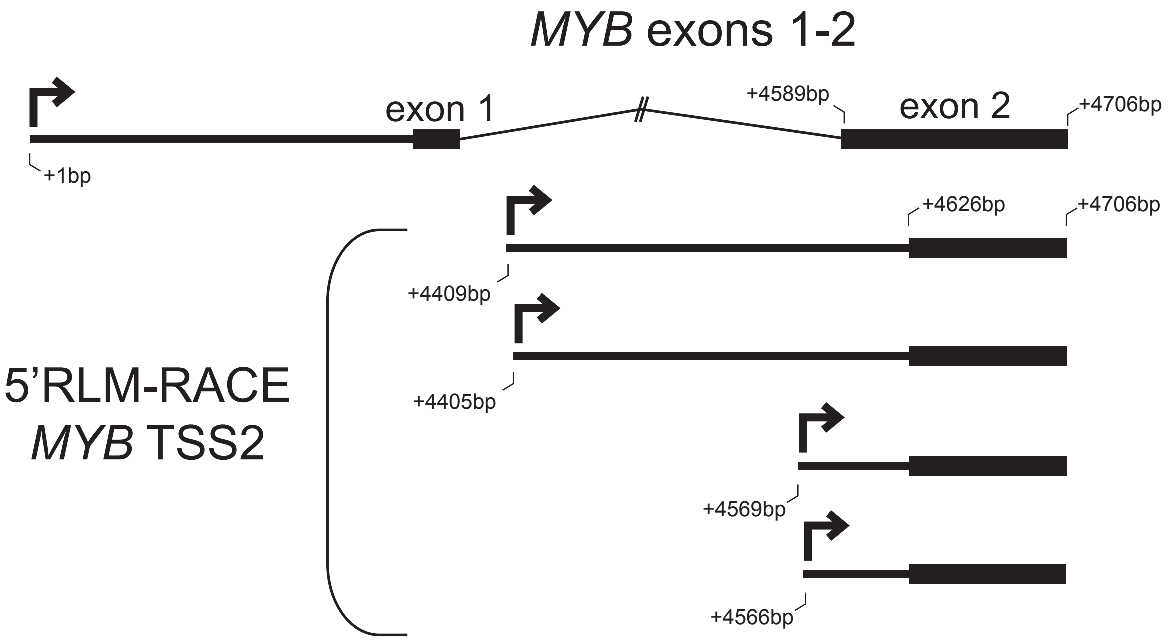

Figure S1. Details of 5' RLM-RACE *MYB* TSS2 products.

MYB TSS2 5'RLM-RACE products were cloned and several colonies were submitted for Sanger sequencing. Importantly, 5'RLM-RACE, as performed here, is not quantitative and does not have single base resolution. Upon sequencing we found transcripts that extended 180 nt, 176 nt, 20 nt and 17 nt upstream of exon 2. We have grouped these into ~180 nt and ~20 nt groups in the main figure. Three colonies from each of the two groups were sequenced.

Figure S2

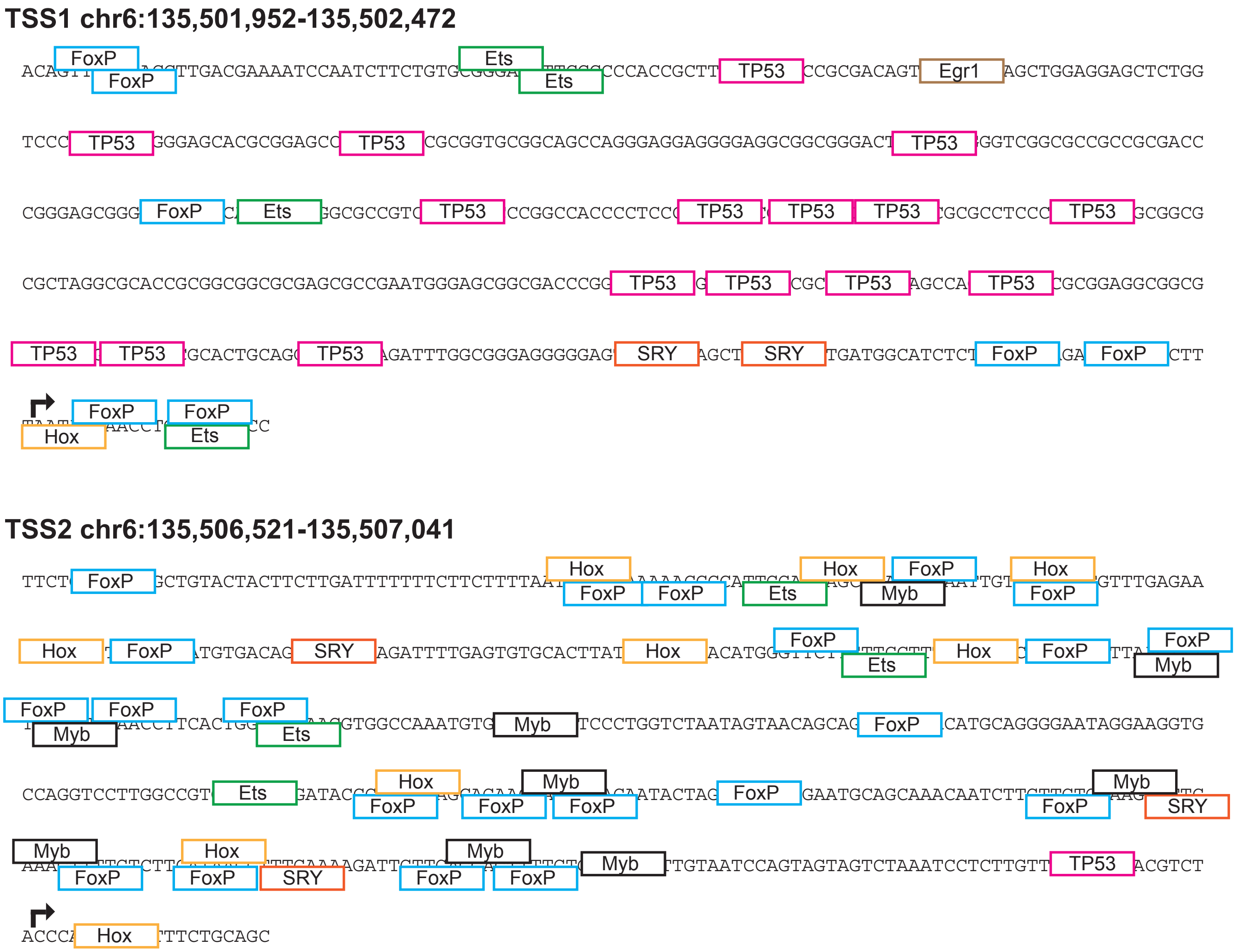

Figure S2. Predicted transcription factor binding sites for the *MYB* promoter reporters. Portions of both *MYB* gene promoters were cloned upstream of the luciferase reporter. Transcription factor binding motifs were discovered using the transcription factor affinity prediction (TRAP) set of web tools (Thomas-Chollier et al. 2011). In ACC tumor RNA-seq data we identified these transcription factor genes that were correlated with MYB expression: EN1, ETV6, FOXO3B, HEY2, MYB, PBX1, SIX3, SOX4, SOX6, SOX8, SOX9, SOX10, SOX11, TP53. The diagrams show the DNA sequences 500 nt upstream of MYB TSS1 and TSS2 promoters, with predicted binding sites for these transcription factors labeled. These binding sites have not yet been confirmed by Chromatin Immunoprecipitation studies.

Figure S3

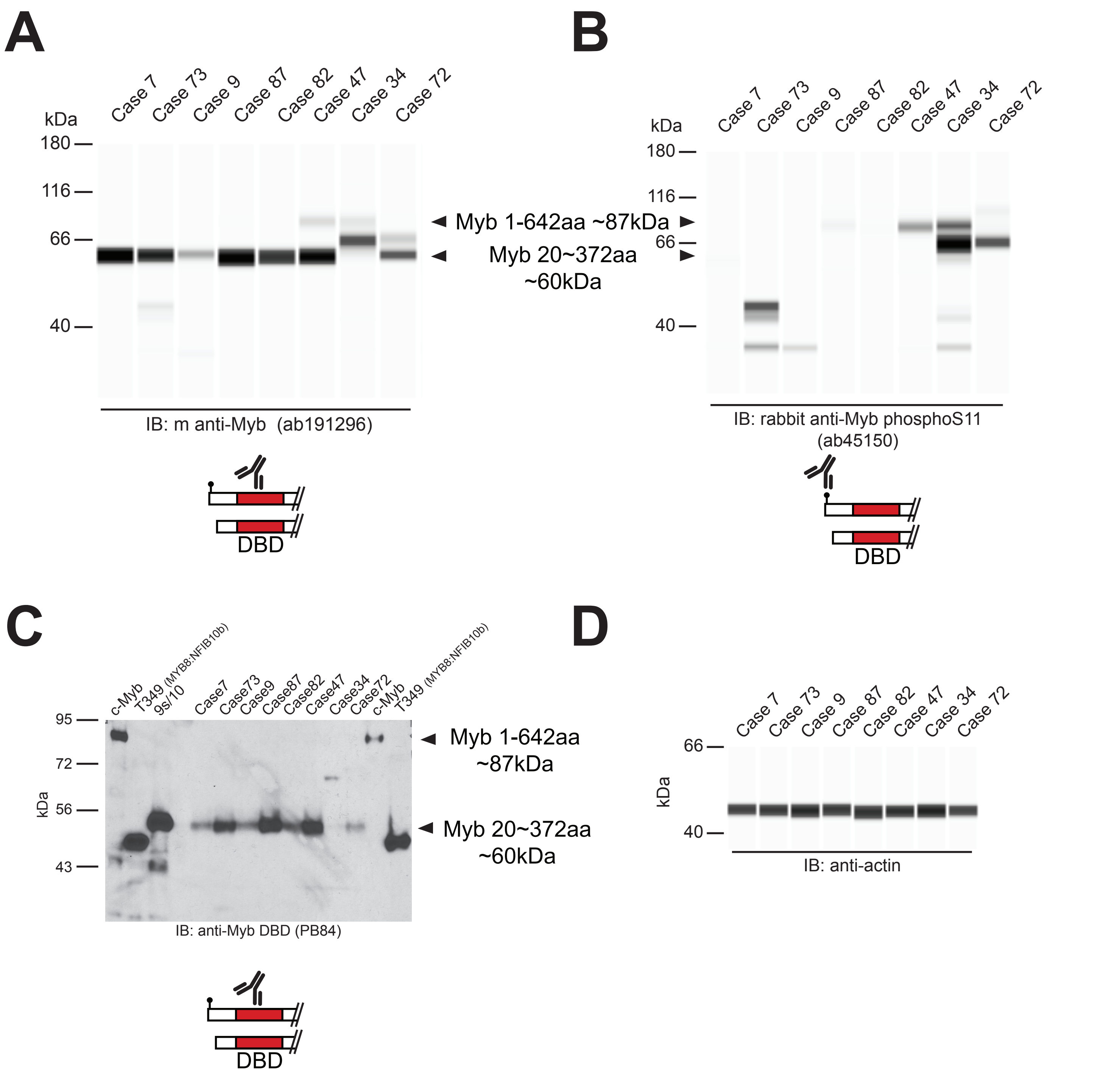

Figure S3. Myb proteins in ACC tumors detected by Western blotting. ACC tumor extracts were analyzed using the WES capillary blotting instruments (panels A, B and D) or by regular Western blotting (C) using antibodies specific for the DNA binding domain (A, C), the N-terminal pS11 motif (B) or actin as a loading control (D). Panel C also shows extracts from HEK293 cells transfected with various Myb expression plasmids, as indicated.

Figure S4

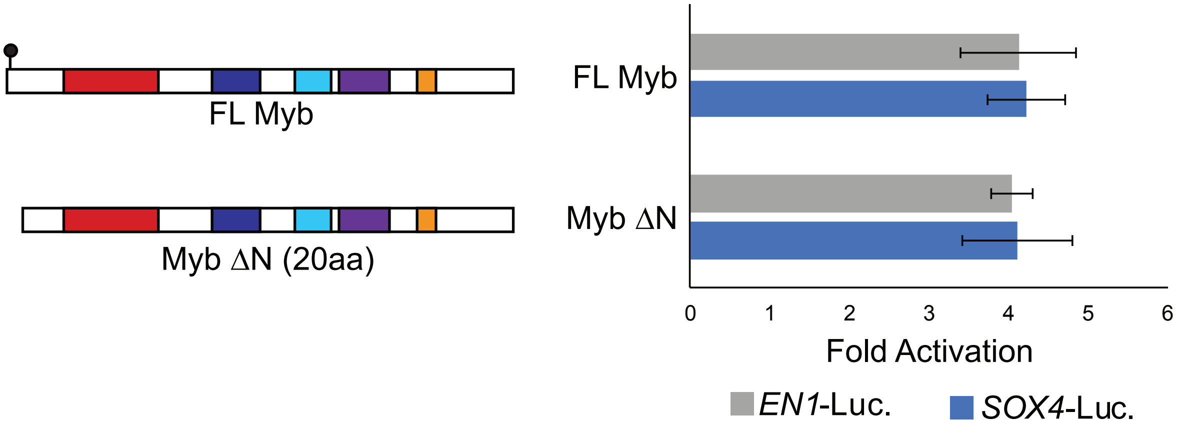

Figure S4. ΔN Myb proteins have similar activities to full-length Myb in reporter assays. Two previously described (Frerich et al. 2018) Myb-responsive reporter genes were used. A portion of the engrailed gene (EN1) promoter and the SOX4 gene promoter were cloned upstream of the firefly luciferase reporter gene. Their response to ΔN Myb and full-length Myb were assayed in HEK293TN cells. ΔN Myb binds and activates expression in a reporter assay and is thus a functional transcription factor.

Figure S5

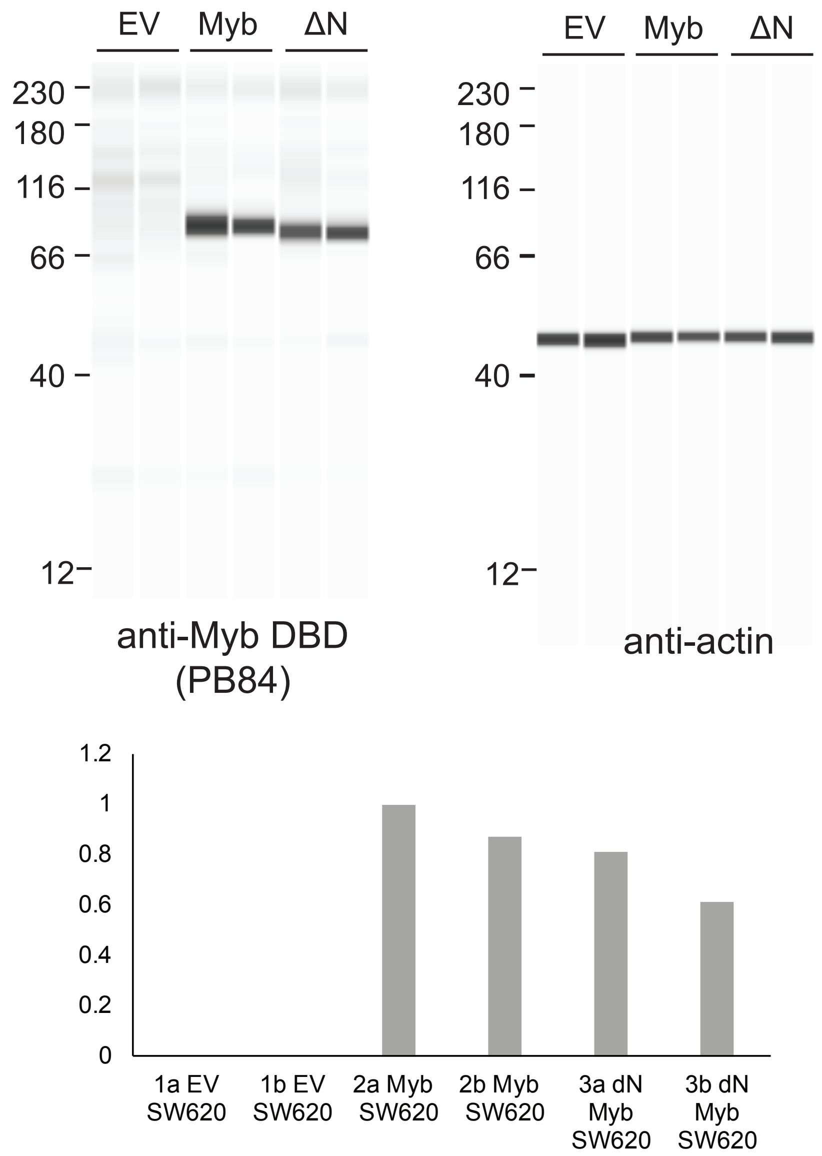

Figure S5. Western blot of ectopically expressed Myb isoforms. SW620 cells were transduced with lentiviral particles to express empty vector, full-length Myb or ΔN Myb. Protein and RNA (used for RNA-seq) were harvested at 48hrs. Western blot analyses were performed using rabbit serum that detects the DNA-binding domain of Myb proteins, and actin was probed as a loading control. Full-length Myb and ΔN Myb were expressed in relatively equal quantities. Protein was quantified relative to actin loading control.

Figure S6

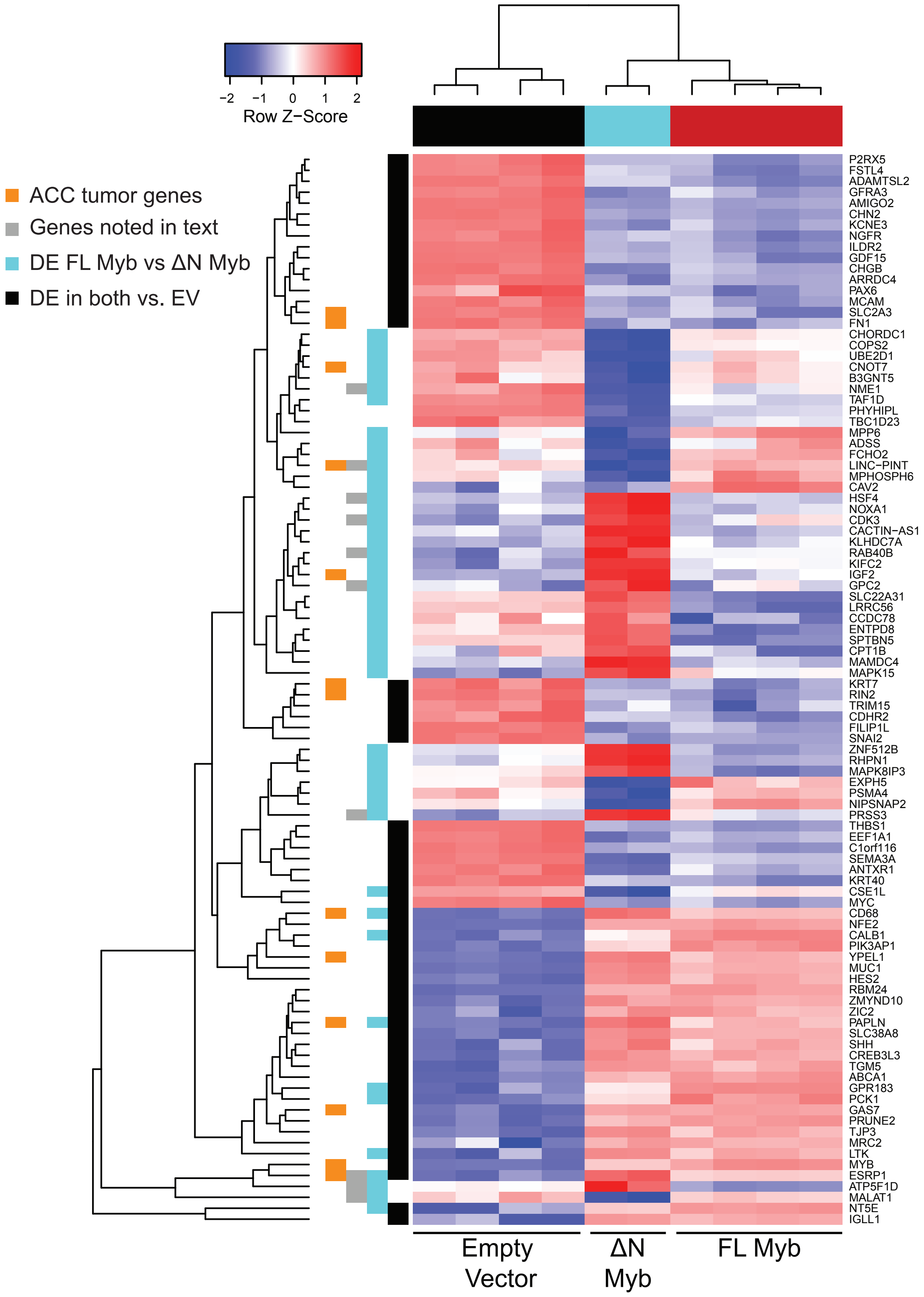

Figure S6. Large heatmap of SW620 RNA-seq. A summary heatmap displays gene expression changes elicited by Myb transcription factors. Color bars along the left side of the heatmap indicate the following: genes differentially expressed in Myb expression ACC tumors versus Myb negative ACC tumors (orange), genes discussed in the text (gray), genes differentially expressed in Myb versus ΔN Myb (cyan), and genes differentially expressed by both Myb and ΔN Myb (black).
